# The Viennese dissection course—A model for Habsburg medical teaching (1787–1848)

**DOI:** 10.1007/s00508-024-02433-2

**Published:** 2024-08-30

**Authors:** Sophia Bauer, Leo Schaukal, Wolfgang J. Weninger

**Affiliations:** 1 Department of Anatomy, Center for Anatomy and Cell Biology, Medical University of Vienna, Vienna, Austria; 2 University of Vienna, Institute of Economic and Social History, Vienna, Austria

**Keywords:** Anatomy, History, Cadaveric dissection, Vienna, 18th and 19th Century

## Abstract

This article delves into the beginnings of the dissection course, a teaching practice which today is still in place in Vienna and continues to shape future medical practitioners. Based on a comparison of different historical sources the article shows that the Viennese tradition of a dissection course dates back to the 1780s and the initiative of the anatomist Joseph Barth to build a dissection institute and to implement a dissection course, two endeavors that coincided with Joseph II’s reform ideas regarding a practically orientated medical and surgical education and a Europe-wide practice turn. Additionally, this paper shows the role of the Viennese dissection course as model for other Habsburg universities and, thus, explains the similarities of today’s dissection courses in different former Habsburg universities.

## Introduction

In the nineteenth century the dissection of human bodies was already a well-established practice in medical teaching at the University of Vienna [1]. As part of the curriculum, with renowned professors as teachers, e.g., the anatomist Joseph Hyrtl (1810–1894) and a (mostly) solid supply of human bodies, the anatomical institute in Vienna was a leading institution when it came to anatomical teaching [2]. There, students of medicine dissected human bodies as part of their education gaining profound and tangible knowledge of the human body. Yet, the history of the development of this teaching practice and its spread across Habsburg universities has not yet been sufficiently analyzed.

There are only a few accounts of a so-called *Sezieranstalt*, a place where students learned human anatomy via dissection, in historical sources dealing with the eighteenth century Viennese anatomy, which, however, do not explicitly mention a dissection course: When Joseph Barth (1745–1818), anatomy professor in Vienna from 1774 to 1791, died in 1818, his former student, professor and ophthalmologist Joseph Beer (1763–1821), mentioned the creation of the *Sezieranstalt* in an epitaph [3]. Decades later in 1869, Joseph Hyrtl referred to the *Sezieranstalt* too but did not mention a dissection course at all [4].

Recent historical research yields similar results: In 2003, Sonia Horn delved into the institutional foundation of the Viennese professorship of anatomy and the anatomical theater, excluding the *Sezieranstalt* or the dissection course [5]. In 2008 Tatjana Buklijas, in her investigation of the history of the acquisition of dead bodies during the eighteenth and nineteenth centuries, did not address this subject either [2]. Also, Erna Lesky, in her work on the second Viennese Medical School (1978), did not cover this anatomical teaching practice [6]. In an anthology dedicated to, amongst others, the history of the Austrian Academy of Science building, published in 2022, the focus was centered on the inauguration of the *Sezieranstalt* in 1787, without mentioning the dissection course at all [7]. Only Markus Oppenauer in 2014, in the context of a new medical curriculum in 1804, briefly mentioned instructions for the dissection course from 1837; however, he did not provide further details [8].

As these examples show, there is still considerable room for research regarding this anatomy teaching practice: Firstly, when was the dissection course originally established in Vienna? Consequently and secondly, bearing in mind that in the eighteenth and first half of the nineteenth century, the university of Vienna, situated in the capital of the Habsburg Empire, served as role model for other imperial universities [9], the following question arises: Did the Viennese dissection course serve as a blueprint for other Habsburg universities? In this context, further (sub)questions emerge: (a) What was the reasoning behind the integration of the dissection course into the medical curriculum? (b) What were the spatial conditions under which the dissection course was conducted? (c) How were the necessary human bodies procured?

We chose the research period between 1787 and 1848 because in 1787 the construction of the *Sezieranstalt* was completed, allowing the dissection course to commence and in 1848 the dissection course was interrupted by the Revolution of 1848, a violent protest spanning, at least in Vienna, from March to December 1848 with demands for the abolition of censorship, the resignation of chancellor Clemens Metternich, a constitution and, on an academic level, the freedom of teaching and learning as central claims, whereafter it was held in other localities [2, 10, 12]. This period was shaped by several medicopolitical reforms, most importantly during Josephinism, a time, in which Joseph II (1741–1790) introduced several reforms focusing, among other things, on social improvements (e.g., the establishment of the General Hospital and institutes for the poor and deaf mutes in Vienna) and education (e.g., the deployment of a collection of wax models open to public at the medical surgical military academy Josephinum), which also emphasized a professionalization of higher medical education (e.g., curricular reforms) [6, 12]. Some of these reforms led to a focus on the practical value of content learned, which strengthened applied knowledge in university teaching. This political development coincided with the foundation of various medical disciplines, e.g., ophthalmology or histology^1^ [6]. The aim of this article is to make a contribution to the research concerning the history of anatomical teaching before 1848. It highlights the history of a teaching method which is still actively being practiced in Vienna today and emphasizes the innovative character of the dissection course in Vienna as a model for other Habsburg universities.

## Material and methods

In this study, we gathered and examined the following archival sources and legal texts concerning the subject of anatomy between 1784 and 1848 and compared: (a) historical building plans^2^, (b) records of the university consistory between 1784 and 1848^3^, available in the Archives of the University of Vienna (UAW) and (c) records of the Court Study Commission (*Studien-Hofcommission*), available at the Austrian State Archives (OeStA) with (d) relevant laws and decrees (between 1784 and 1848) [13, 14].

However, there were certain limitations to our investigation: In the archival sources, for example, the number of bodies used for the dissection course and the lecture was not evident. Also, when it comes to the practices used in and for the dissection course, we had to rely on fragmented pieces of information throughout longer periods of time, as there are only few accounts available. Still, they provided an insightful picture of the early dissection course in Vienna. Yet, detailed answers to why or how Joseph Barth came up with the idea of a dissection course are not possible due to a lack of relevant source material.

### The European practice turn in eighteenth century medicine—A changing Viennese medical curriculum

European medicine changed significantly in the eighteenth century as it adopted a more practically oriented approach: Michel Foucault mentions the example of changes in patient treatment from home-based to hospital-based and new instruments of clinical practice at the center of changes in France [15]. Also, in 1726, the Prussian king Friedrich Wilhelm I ordered the foundation of a dissection course in Berlin, where students of the medicosurgical college (not the university) could dissect bodies themselves [16].

**Fig. 1:**
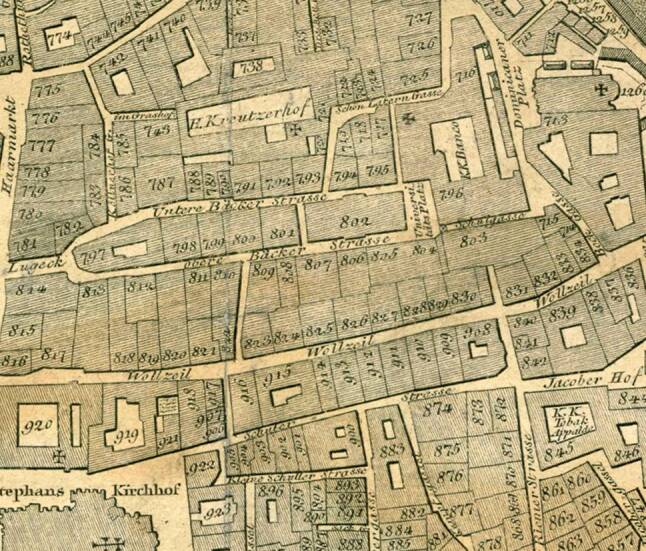
Location of the old university building in Vienna; Grimm, Max von, and Hieronymus Benedicti. Grundriss Der K.K. Haupt- Und Residenzstadt Wien mit Ihren Vorstädten. Artaria Comp., 1810

**Fig. 2:**
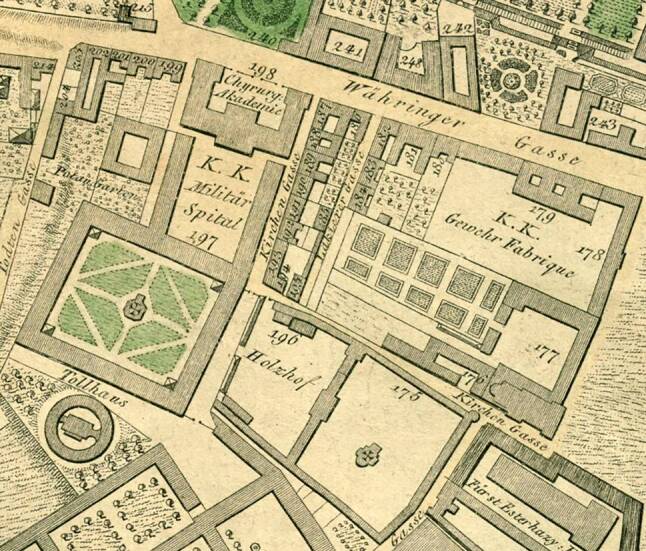
Location of the Josephinum in Alservorstadt; Grimm, Max von, and Hieronymus Benedicti. Grundriss Der K.K. Haupt- Und Residenzstadt Wien mit Ihren Vorstädten. Artaria Comp., 1810

In Vienna, similar developments took place: In 1757, a new university building that housed modern facilities for teaching was opened by Maria Theresia (1717–1780) and her husband Franz Stephan von Lothringen (1708–1765) in Vienna. (Figure 1) Also, in 1785 Joseph II (1741–1790) founded the Josephinum, a military medico-surgical academy, which fostered the practical skills of future military doctors ([17]; Fig. 2). This was part of a new concept of a healthy public body, based on the idea of the citizens’ well-being as a state resource [12, 18].

Anatomy played a leading role in the Austrian practice turn supported by the Emperor. Although the new university building from 1757 included an anatomical theater and a dissection room (not to be confused with the later *Sezieranstalt*), after more than 20 years, changes were made again, corresponding with international developments and Joseph II’s aim to focus more on practical than theoretical knowledge. [7, 14, 19].

In 1784, the construction of a *Sezieranstalt*, based on anatomist Joseph Barth’s (1745–1818) ideas, began and was completed in 1787 [7]. The project was dear to Barth, who as the emperor’s personal ophthalmologist had direct access to the monarch and presumably had the opportunity to use this access to influence imperial decision making in his favor [3]. But concerning the development of his idea, the available sources did not indicate from whom or from where Barth got the idea to build the *Sezieranstalt*. In 1786, Joseph II soon picked up the architectural changes proposed by Joseph Barth in a discussion of a new medical curriculum (and a new order of subjects) with the professorial collegium that was supposed to suit the needs of the time and introduced an obligatory dissection course [6]: the Emperor himself wanted every future medical professional (surgeons and doctors) to learn about the construction of the body (*Bau des menschlichen Körpers*) by dissecting bodies over a period of 6 months.^4^ The gain of basic anatomical knowledge was intended to help with other medical subjects.^5^ In the same year, Joseph II decreed the new curriculum, implementing these changes, including the introduction of a mandatory dissection course, which was to be held by a prosector [14, 19].

After 1786 with respect to the subject of anatomy, additional changes were made that did not affect the *Sezieranstalt* or course. In fact, they supported the macroscopic anatomical approach: in February 1804 a new curriculum for medical studies was published that confirmed the joint curriculum of medicine and surgery and thus, the dissection course. Anatomy still was to be taught in the first year combined with a dissection course [20]. In 1810, after a prosector had been responsible for macroscopic anatomy since 1786, it was ultimately turned into a full professorship with Aloys Michael Mayer (1766–1831), former prosector between 1800 and 1810, as professor [6]. This order of studies was maintained until after the outbreak of the revolution in 1848 [19].

### The Viennese *Sezieranstalt* and dissection course—A Habsburg model?

**Fig. 3:**
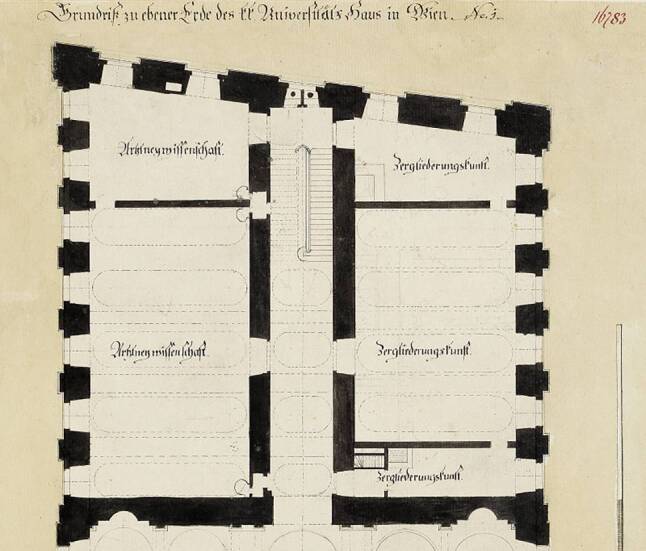
Part of a sketch by Johann Georg Mack, 1783, copper engraving cabinet of the Academy of Fine Arts Vienna, Inventory No. HZ-16.783. Dissection hall (“*Zergliederungskunst*”) on the right hand side

**Fig. 4:**
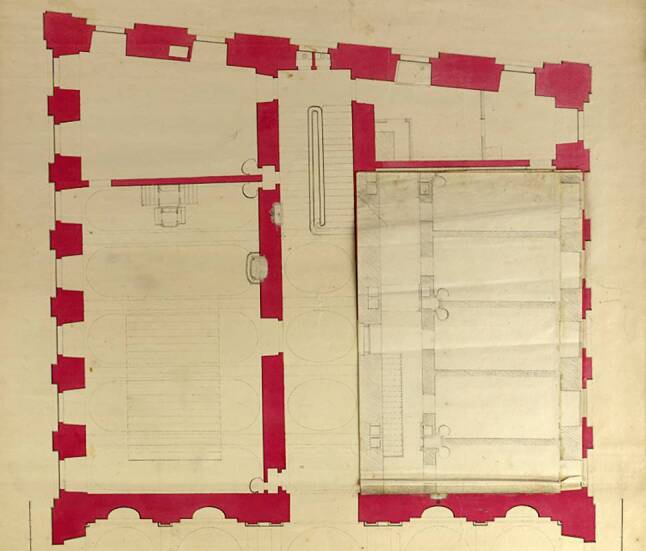
Part of the ground floor after the modifications made in 1784, Albertina, Vienna, Inventory No. AZ8029 (M45/U5/No. 13). *Sezieranstalt* with five dissection rooms on the right hand side

According to the building plans (Figs. 3 and 4) the Viennese *Sezieranstalt* was situated in the back of the ground floor (*Parterre*) of the old university building. It consisted of six separate rooms, including five dissection rooms and one vestibule (*Prosektur*). At the back there was also an elevator (*Leichenlift*) capable of lifting newly arrived bodies from the basement to the ground floor and a well for cleaning purposes. For teaching purposes, next to the *Sezieranstalt* there was also an anatomical museum containing a large number of anatomical exhibits: Petra Aigner and Stefan Sienell estimated a total number of ca. 2000 for the period until 1848 [7]. These included human anatomical and pathological remains (embalmed, wet, dry and injected specimens) depicting the shape of the body and natural rarities (“*Naturseltenheiten*”^6^) [8].

Concerning the dissection course, according to a decree from the Court Study Commission from 12 October 1810, each student had to pay a fee contributing to the costs of the body transportation [13]. In 1836/1837, this practice was shut down in Vienna by the commission that ordered the Study Fund (“*Studienfonds*”) to pay for bodies for dissection. Also, all anatomy professors in the Empire were ordered to personally supervise the dissection course [8, 13]. Regarding the syllabus of the course, the primary sources provide little information; however, in the foreword of his dissecting instructions, published in 1822 Mayer, since 1805 as *prosector legens* (a prosector, who was, next to dissecting bodies during the lectures, also allowed to teach students himself)^7^, and since 1810 as professor responsible for macroscopic anatomy [4, 21, 22], described the dissection course as a self-practice, which takes place in the university’s dissection room (“*Zergliederungssaal *(*Sekziersaal*) [sic!]”), together with a rich supply of bodies [23].

With respect to the content of the dissection course, Mayer mentioned that the students had to expose the muscles and ligaments, reveal the sensory organs, the joints, the nerves, inner organs, the sexual organs (male and female) and dissect them. Mayer stated that using male bodies only would have been the “ideal” situation because the muscles would be bigger and easier to grasp and see. Yet, if not otherwise possible, he agreed to dissecting females, which implies that students came into contact with bodies of both sexes [23]. In his article Markus Oppenauer cites a decree, which in 1837, Joseph Berres (1796–1844) was then professor of anatomy [21, 22], ordered students in Vienna to dissect all muscles, the vascular and nervous systems as well as the internal organs and to make obligatory anatomical specimens (“*Pflichtpräparate*”). Outstanding specimens were integrated into the anatomical museum [8]. This decree further emphasized the dissection program which Mayer had already introduced.

The already mentioned decree from 1810, which introduced a new medicosurgical curriculum, also instructed all country offices (“*Länderstellen*”) that a *Sezieranstalt* had to be installed at every university and lyceum (a “proto”-university without the rights of university faculties, where philosophy, later after some Habsburg universities, e.g., in Lemberg (Lviv), were degraded to lyceums, also medicine, was mainly taught as preparation for further university studies in law, medicine or theology [24]) in the Empire. Also, at every university and lyceum in the Empire bodies had to be dissected in the presence of the professor and the students [13]. Thus, the decree chose the *Sezieranstalt* and processes already established in Vienna as a template for other universities in the Habsburg Empire. This Empire-wide introduction might have been the result of visible positive impacts of the introduction of the dissection course as it strengthened the quality of the medical education, especially the practical skills. Overall, this placed the emphasis on the practical value of content introduced with Josephinian university policy. Thus, it seems that when the Viennese *Sezieranstalt* and dissection course had been proven to be a valuable addition over decades, they became a blueprint for other Habsburg universities.

After 1836 no further changes were made to the dissection course until 1848, when the revolution interrupted the course for almost a year. Afterwards, anatomy and other medical disciplines were relocated to new institutes and buildings dispersed across Vienna [11].

### Dead bodies—The supply of bodies at the Viennese medical faculty from 1749 until after the revolution of 1848

With respect to the supply of bodies, the rules installed by Maria Theresia, allowing the bodies of deceased hospital patients to be used for dissection, prevented a lack of bodies – a threat for anatomical teaching, which, for example, in Cambridge had led to the absence of a professor of anatomy and his dismissal in 1728 [25]. In 1749, Maria Theresia decreed that due to a shortage of bodies of executed criminals (the year before only three persons were executed) bodies of patients who had died in hospital could be used for anatomy education at the university [2, 5]. With this decree, the supply of bodies for anatomical dissection was legally secured for the next decades. This decree also led to additional human bodies being used in the newly established dissection course in Vienna. Later, in a decree from 1810 the usage of bodies from hospitals was universally implemented for all Habsburg lyceums and universities [13].

In Vienna, different hospitals provided the university with bodies: From 1787 until 1848, bodies were supplied by the General Hospital (*AKH*) and the Hospital of the Brothers of St. John of God (*Barmherzige Brüder*)^8^ [2, 8]. Between 1845 and 1848 the Military Hospital became an additional source [2].

What precisely happened to those bodies after death? When patients died or criminals were executed, at least in 1834, no later than 3 days after their passing the bodies were sent to the university building.^9^ There they were stored in tubs, filled with ice cold water.^10^ The water was changed from time to time to prevent the smell of decay.^11^ When finally needed for the dissection course, the bodies were moved to the vestibule with the body elevator (*Leichenlift*) [7]. If parts of the body were found suitable for the anatomical museum, they were preserved (through maceration, injection with fluids or preservation in ethyl alcohol (*Weingeist*)) and then integrated in the anatomy collection [23].

However, this system of body acquisition collapsed with the revolution. Not only was the anatomy institute moved to the Josephinum after the revolution but also new resources were required for maintaining the dissection course and anatomy teaching as a whole because the Military Hospital had ended its collaboration with the anatomy institute, leading to a lack of bodies [2]. During this time the anatomy professor Joseph Hyrtl established new ties with a hospital in the Viennese suburb Wieden^12^, securing a new source for dead bodies. He also petitioned the Ministry for Internal Affairs (*k.k. Innenministerium*) for bodies of deceased prisoners from different jails (the *Provinzial-Strafhaus* in Leopoldstadt and the prison hospital in Josephstadt), to counter the lack of bodies. Apparently, the original tradition of using the bodies of deceased prisoners dating back to Maria Theresia had ceased at some point. After some hesitation the ministry granted Hyrtl’s request in 1853.^13^

## Discussion

This paper uncovered that the dissection course was introduced by Joseph II in 1786/1787, based on the ideas of Joseph Barth and the newly emphasized practical value of content. Only a few decades later this curriculum was then instituted at other Habsburg universities and lyceums. In Vienna, the course was located in purpose-built dissection halls in the old university building. Unfortunately, more details on the spatial conditions (number of students and bodies, actual size of the *Sezieranstalt* etc.) could not be answered with the available sources. The research revealed that the *Sezieranstalt* received human remains from the General Hospital and the Hospital of the Brothers of Saint John of God, both based in former suburbs of Vienna. Additionally, the research uncovered (a) precisely who and which regions of the body were dissected in the dissection courses, (b) which contents were taught, at least during Mayer’s and Berres’ professorships and (c) how human bodies were used in the context of dissection.

In the eighteenth and the first half of the nineteenth century, according to Jan Surman, the university of Vienna in general acted as a role model for other imperial universities [9]. As has been seen, the dissection course was another part of this Viennese position as a forerunner for other Habsburg universities. The effects are showcased by Tatjana Buklijas, who, in the introduction of her PhD thesis, stated that in the 1990s the anatomical teaching practices she experienced in Zagreb were still very similar to Vienna and that the similarity stemmed from the Viennese school of medicine [26].

This paper not only highlighted the remarks of Surman and Buklijas of Vienna as a model for medical teaching but also showed that they are equally applicable to the history of the dissection course: With respect to the practice turn, the curricular changes, especially the introduction of the dissection course in 1786/1787, applied to the medical curriculum in the 1780s were originally introduced in Vienna and consequently, in 1810, to all Habsburg universities and lyceums.

In conclusion and within a broader European context, this article revealed that Vienna is an earlier example of a university that installed a dissection course (1786/1787) as part of this Europe-wide practice turn. For example, in Berlin, Edinburgh and Belgium dissection courses were introduced at universities in 1810, 1833 and 1876, respectively [16, 27, 28]. Yet, some universities like Cambridge (1716/1717) or Paris (1732/1733) are even earlier examples than Vienna [29, 30]. Thus, proving that the Viennese course, and more generally the pan-Habsburg development, were part of a broader and long-term European process. This research showed the limitations regarding the source material and secondary literature. Consequently, this study left some questions unanswered and could only hypothesize in other cases.

## Conclusion

There are several questions which need further research: Why did Joseph Barth come up with the idea of a *Sezieranstalt* in the first place? How exactly did he convince Joseph II of his idea? What was taught in the dissection courses during the first decades of their existence? What were the exact operational processes? What were the processes behind the establishment of dissection courses at other European universities (e.g., Cambridge or Paris)? If further research were available on the introduction of human dissection courses at other Habsburg or European universities, it could potentially answer some of the open questions by providing additional source material not available in this study. Although some of the secondary literature provides information on when exactly dissection courses were introduced in Cambridge or Paris, it excludes the main protagonists, ideas and discussions, operational procedures and institutional frameworks involved in this process.

It was hypothesized that the Viennese dissection course served as a blueprint for other Habsburg universities and lyceums, which this study proved to be true. Showing the spatial conditions of the dissection course, contents taught therein as well as the political framework, this article provides the future opportunity to conduct comparative studies between Vienna and other Habsburg as well as European universities or lyceums regarding the introduction of *Sezieranstalten* and courses in more detail, thus contributing to a widened understanding of the distribution and formation of medical teaching practices across the Habsburg Empire and Europe as a whole.

## Footnotes

1 Note: Known to contemporaries as ‘microscopic anatomy’.

2 Note: Albertina, Vienna, Inventory No. AZ8029 (M45/U5/No. 13) (1784), published in “The Austrian Academy of Sciences 1847–2022” and Copper Engraving Cabinet of the Academy of Fine Arts Vienna, Inventory No. HZ-16.783 (1783).

3 Note: In the “Consistorial-Akten” (consistory files) at the UAW, in regards to the renewal of the “Sezieranstalt”, architectural sketches and descriptions of the building site were mentioned that were sent to the government of Lower-Austria. But a research-based request sent to the Allgemeines Verwaltungsarchiv (AVA), which is part of the ÖStA, and the Archive’s answer showed that they burnt in a fire that destroyed the “palace of justice” in Vienna in 1927. Thus, we could only look at the correspondence present in both archives and not the sketches.

4 AT-OeStA/AVA Unterricht StHK Teil 1 A 18.1, Akt 169 ex 1786, fol. 22, verso (24. Mai 1786) and AT-OeStA/AVA Unterricht StHK Teil 1 A 18.1, Akt 169 ex 1786, fol. 25, recto (June 17th, 1786).

5 AT-OeStA/AVA Unterricht StHK Teil 1 A 18.1, Akt 169 ex 1786, fol. 22, verso (May 24th, 1786).

6 AT-OeStA/AVA Unterricht StHK Teil 1 A 18.1, Akt: 4 Med, Präparatensammlung, anatomische und pathologische. 1789, fol. 2, recto (July 1st, 1789).

7 Note: Mayer became prosector in 1800, but lectured macroscopic anatomy since 1805, when he became the successor of the prosector legens Joseph Ehrenritter.

8 AT-UAW/CA 1.3.529, “Geruchsbelästigung durch die anatomischen Übungen in der Universität” (20.06.1833–28.10.1835).

9 Ibid.

10 AT-UAW/CA 1.3.245, “Beheizung der Wohnung des Prosectors anatomiae” (13.01.1811–15.01.1811).

11 AT-UAW/CA 1.3.529, “Geruchsbelästigung durch die anatomischen Übungen in der Universität” (20.06.1833–28.10.1835).

12 AT-OeStA/AVA, Unterricht Allgemein (1848–1940), Ktn. 845, Sign. 4 G Med, Nr. 8242/641, fol. 4.

13 Ibid., fol. 1 recto and fol. 4, and AT-OeStA/AVA, Unterricht Allgemein (1848–1940), Ktn. 845, Sign. 4 G Med, Nr. 3129/180, fol. 9 recto et verso.

## References

[1] Lohff B. Gedanken zum Begriff Wiener Medizinische Schule. In: Angetter D, Nemec B, Posch H, Druml C, Weindling P, editors. Strukturen und Netzwerke Medizin und Wissenschaft in Wien 1848–1955. Göttingen: V&R unipress GmbH; 2018. pp. 41–72.

[2] Buklijas T. Cultures of death and politics of corpse supply: anatomy in Vienna, 1848–1914. Bull Hist Med. 2008;82(3):570–607. 18791297 10.1353/bhm.0.0086 PMC2633446

[3] Beer J. Barth’s Nekrolog. Erneuerte vaterländische Blätter für den österreichischen Kaiserstaat. 1818. pp. 133–6.

[4] Hyrtl J. Vergangenheit und Gegenwart des Museums für menschliche Anatomie an der Wiener Universität. Wien: Braumüller; 1869.

[5] Horn S, „ ein wohl auffgerichtes theatrum anatomicum. Anatomischer Unterricht für nichtakademische Heilkundige an der Wiener Medizinischen Fakultät im 18. Jahrhundert. In: Stukenbrock K, Helm J, editors. Anatomie Sektionen einer medizinischen Wissenschaft im 18 Jahrhundert. Stuttgart: Steiner Verlag; 2003. pp. 189–212.

[6] Lesky E. Die Wiener Medizinische Schule im 19. Jahrhundert. 2nd ed. Graz: Böhlau Verlag; 1978.

[7] Aigner P, Sienell S. Nutzungsgeschichte des Akademiegebäudes (1857–2022). In: Feichtinger J, Mazohl B, editors. Die Österreichische Akademie der Wissenschaften 1847–2022: eine neue Akademiegeschichte. Wien: Verlag der österreichischen Akademie der Wissenschaften; 2022. pp. 205–46. Denkschriften der Gesamtakademie / Österreichische Akademie der Wissenschaften 88; vol. 3.

[8] Oppenauer M. Solche Asyle sind die Sammlungen und Museen, welche die Gegenwart der Wissenschaft darstellen, und ihre Zukunft vorbereiten.“ Soziale Aspekte der Anatomie und ihrer Sammlungen an der Wiener Medizinischen Fakultät, 1790–1840. Sudhoffs Arch. 2014;98(1):47–75. 25007447

[9] Surman J. Universities in imperial Austria 1848–1918. A social history of an academic space. West Lafayette: Purdue University Press; 2019.

[10] Maisel T. Lehr- und Lernfreiheit und die ersten Schritte zu einer Universitäts- und Studienreform im Revolutionsjahr 1848. In: Aichner C, Mazohl B, editors. Die Thun-Hohenstein’schen Universitätsreformen 1849–1860: Konzeption – Umsetzung – Nachwirkungen. Wien: Köln/Weimar: Böhlau Verlag; 2017. pp. 99–117.

[11] Buklijas T. Eine Kartierung anatomischer Sammlungen im Wien des 19. Jahrhunderts. In: Angetter D, Nemec B, Posch H, Druml C, Weindling P, editors. Strukturen und Netzwerke Medizin und Wissenschaft in Wien 1848–1955. Wien: V&R unipress; 2018. pp. 97–116.

[12] Vocelka K. Geschichte Österreichs. Kultur – Gesellschaft – Politik. 8th ed. München: Heyne Verlag; 2002.

[13] Thaa G. Sammlung der für die österreichischen Universitäten giltigen Gestze und Verordnungen. Wien: Verlag der G. J. Manz’schen Buchhandlung; 1871.

[14] Kink R. Geschichte der kaiserlichen Universität zu Wien. Erster Band, Geschichtliche Darstellung der Entstehung und Entwicklung der Universität bis zur Neuzeit, 1. Theil, Geschichtliche Darstellung, sammt urkundlichen Beilagen. Vol. 1. Wien: Gerold; 1854.

[15] Foucault M. Die Geburt der Klinik. Eine Archäologie des ärztlichen Blicks. 10th ed. Frankfurt am Main: S. Fischer; 2016.

[16] Winkelmann A. Sezieren und Sammeln. 300 Jahre Berliner Anatomie 1713 bis heute. In: Beddies T, Schmiedebach HP, editors. Hefte zur Geschichte der Charité – Universitätsmedizin Berlin. Berlin: be.bra Wissenschaft Verlag; 2018.

[17] Lohff B. Die Josephs-Akademie im Wiener Josephinum. Die medizinisch-chirurgische Militärakademie im Spannungsfeld von Wissenschaft und Politik 1785–1874. Böhlau Verlag; 2019.

[18] Spary EC. Health and medicine in the enlightenment. In: Jackson M, editor. The Oxford handbook of the history of medicine. New York: Oxford University Press; 2011. pp. 82–99.

[19] Angetter D. „Die Tiefen der Medizin bleiben also denjenigen verborgen, die die Naturgeschichte nicht kennen“. Studienordnungen, Universitätsreformen und Fragen nach dem Wert eines geistes- und naturwissenschaftlichen Grundlagenwissens für das Medizinstudium. In: Angetter D, Nemec B, Posch H, Druml C, Weindling P, editors. Strukturen und Netzwerke Medizin und Wissenschaft in Wien 1848–1955. Göttingen: V&R unipress; 2018. pp. 155–78.

[20] -. Plan zu einer gleichmäßigen auf allen Universitäten der österreichischen Monarchie zu beobachtenden Studienordnung, in Bezug auf Arzneykunde, Wundarzneykunst und Pharmacie. Med Zeitung. 1804;2(36):193–204.

[21] Bauer S. Ein anatomischer (Lehr‑)Körper. Symbolisches Kapital und Disziplingründung anhand der Wiener Lehrstuhlinhaber für Anatomie (1735 bis 1844) [Master Thesis. Wien: University of Vienna; 2021.

[22] Bauer S, Schaukal LM, Weninger WJ. The influence of censorship laws on viennese anatomy textbooks from the outgoing 18th century until after the student revolution of 1848 in Austrian absolutism. Anat Anz. 2023;250:152129. 10.1016/j.aanat.2023.152129 37467810

[23] Mayer AM. Praktische Anleitung zur Zergliederung des menschlichen Körpers. Ein Hülfsbuch bey anatomischen Übungen für seine Schüler. Wien: Carl Ferdinand Beck; 1822.

[24] Ptashnyk S. Sprachengebrauch und Sprachenwechsel an der Lemberger Universität im ausgehenden 18. und in der ersten Hälfte des 19. Jahrhunderts. In: Vernakuläre Wissenschaftskommunikation. Berlin/Boston: Walter de Gruyter; 2018. pp. 335–60.

[25] Kaufman MH. 500 years of the college of surgeons and 300 years of the chair of anatomy in edinburgh. Surgeon. 2005;3(3):234–41. 16076010 10.1016/s1479-666x(05)80046-0

[26] Buklijas T. Dissection, discipline and Urban transformation. Anatomy at the university of Vienna, 1845–1914 [Diss. Cambridge: Clare Hall College; 2005.

[27] Struthers J. Historical sketch of the edinburgh anatomical school. Edinb Med J. 1866;12(6):539–59. 29646308 PMC5311681

[28] Claes T. Corpses in Belgian anatomy, 1860–1914: nobody’s dead. Cham: Palgrave MacMillan; 2019.

[29] Brierley C, Human Stories & Ideas. Body of work. The silent teacher helping students learn anatomy. 2016. https://cambridge-uni.medium.com/body-of-work-the-silent-teacher-helping-students-learn-anatomy-2985c0cb84ab. Accessed 26 June 2024.

[30] Gelfand TT. “Paris manner” of dissection. Student anatomical dissection in early eighteenth-century Paris. Bull Hist Med. 1972;46(2):99–130. 4564766

